# Diagnostic Accuracy of Quick Stick for Identifying Traumatic Patients in Need of Tetanus Prophylaxis; a Cross-sectional Study

**Published:** 2017-04-30

**Authors:** Iraj Golikhatir, Seyed Hossein Montazer, Nabiollah Bagheri, Fatemeh Jahanian, Farzad Bozorgi, Seyed Mohammad Hosseininejad, Hamed Amini Ahidashti

**Affiliations:** 1 Emergency Department, Imam Khomeini Hospital, Mazandaran University of Medical Sciences, Sari, Iran.

**Keywords:** Tetanus, point-of-care testing, enzyme-linked immunosorbent assay, immunoglobulins, prevention and control, wounds and injuries, emergency service, hospital

## Abstract

**Introduction:**

Based on the existing studies, measuring serum level of immunoglobulin for making decisions regarding prescription of tetanus prophylaxis seems logical and cost effective. Therefore, the present study was done with the aim of evaluating the diagnostic accuracy of tetanus quick stick (TQS) in comparison with ELISA method in this regard.

**Methods:**

The present diagnostic accuracy study was carried out on trauma patients presenting to emergency department, who were in need of receiving tetanus prophylaxis due to dirty wounds or injuries. Patients’ blood was evaluated regarding presence of anti-tetanus antibody via TQS and ELISA methods and screening performance characteristics of TQS in identifying the cases in need of receiving prophylaxis was calculated compared to ELISA as the reference test.

**Results:**

148 patients with the mean age of 34.58 ± 15.86 years (4-86) were studied (87.8% male). Agreement rate between the results of TQS and ELISA was 0.78 based on calculation of kappa coefficient. Sensitivity, specificity and area under the ROC curve of TQS were estimated to be 100 (95% CI: 96.50 – 100), 66.66 (95% CI: 38.68 – 86.01), and 0.83 (95% CI: 0.68 – 0.98), respectively. If TQS was used, the cost of treatment regarding use of tetabulin could have a 91.7% reduction.

**Conclusion:**

Based on the findings of the present study, TQS has good diagnostic accuracy in comparison with ELISA and considering its 100% sensitivity and negative predictive value in cases with dirty wound, it can be considered as a reliable tool for screening patients that do not need to receive anti-tetanus prophylaxis.

## Introduction

Tetanus manifests in all age groups and geographical areas after a wound comes into contact with anaerobic Gram positive bacteria called Clostridium tetani (1-7). The risk of developing the disease is higher in hot and humid areas, injecting drug addicts, people who have not been vaccinated, and those with a deficiency in their immune system. Prevalence of tetanus in the developing countries is 135 times more than that of developed countries and its death rate has been estimated to be about 20% to 45% in those affected (8). Currently, in many emergency departments (EDs) making decisions regarding the need for tetanus prophylaxis prescription is done based on vaccination history and wound characteristics (9-11). However, noting that the patients do not provide reliable history regarding vaccination, using serum immunoglobulin level measurements for making decisions regarding prescription of tetanus prophylaxis seems to be more logical and cost effective (6, 12-17). 

Tetanus quick stick (TQS) is a tool for qualitative measurement of immunoglobulin via immunochromatographic assay and its use increases reliability of vaccination history (6, 15, 18). Yet, various opinions exist regarding the screening performance characteristics of this test and its sensitivity and specificity have been estimated to be about 76 to 88% and 97 to 100%, respectively (6, 18, 19). The present study was done with the aim of evaluating the diagnostic accuracy of TQS in determining the condition of serum level of tetanus immunoglobulin in comparison to ELISA method.

## Methods

***Study design***

The present diagnostic accuracy study was carried out on trauma patients presenting to ED of Imam Khomeini Hospital, Sari, Mazandaran, Iran, throughout the time between October 2015 and November 2016, who were in need of receiving tetanus prophylaxis due to wounds or injuries. Protocol of this study was approved by the ethics committee of Mazandaran University of Medical Sciences after evaluation in the research council of emergency medicine specialists group. To maintain confidentiality of patients’ medical profile data and adhering to ethical practice, the researchers keenly adhered to the principles introduced in the declaration of Helsinki during the study period. Information regarding the study method was given to the participants and written consent was obtained from them before being included in the study. No treatment intervention was done in the study and all the costs of the project were covered by the researchers.

***Participants***

Trauma patients presenting to the mentioned ED with dirty wounds or injuries (tetanus prone), who needed tetanus prophylaxis were evaluated using non-probability convenience sampling during one year, until the required sample size was reached. Patients with severely bleeding wounds, those in need of rapid care with surgery, and pregnant women were excluded from the study.

In this study, a wound made more than 6 hours before, contaminated with soil or saliva, caused by puncture (nail going in), compression, bullet, burn, and frostbite were considered as dirty wound. Complete vaccination was defined as history of more than 3 shots of tetanus vaccine and incomplete or undefined vaccination was history of 3 times or less injections. 

***Data gathering***

A senior emergency medicine resident was responsible for gathering data of the patients by completing a pre-designed checklist including baseline characteristics (age, sex, vaccination history), type of prophylaxis prescribed in ED (tetabulin, vaccine, none) as well as results of patients’ serum evaluation regarding presence of anti-tetanus antibody using TQS and ELISA. Two separate individuals performed TQS test and ELISA and were blind to the results of the other test.

***TQS test method***

After gathering preliminary data, TQS test was done on the patient’s bedside by a trained nurse or physician in charge of the patient. A drop of blood from the patient’s fingertip was placed on the TQS kit (made in China) and 3 drops of the corresponding buffer was added to it. There were 2 marks on the kit, the C (control) and T (tetanus) lines. After adding the buffer, a maximum of 10 minutes was given for the lines to change color. In this kit color change of the C region indicated correct sampling and color change in the T region showed presence of tetanus antibody (in case of not being immune to tetanus, no color change will be seen in this region). No change of color in the C region indicated an error and in this case, the test was repeated with another kit.

***ELISA method***

ELISA was used as the reference test to determine serum level of anti-tetanus IgG. 2.5-3 cc of the patient’s venous blood was drawn by a trained technician and immediately sent to the laboratory. If the level of this antibody was higher than 0.5 IU/ml, immunity against tetanus was positive and there was no need for prophylaxis against it. If the antibody level was lower than 0.1 IU/ml, immunity to tetanus was negative and there was need for prophylaxis.

***Statistical analysis***

Required sample size for performing the present study considering the 74.1% prevalence of immunity to tetanus, type 1 error of 5%, type 2 error of 10%, and need for immunity of 56.9% was calculated to be 148 cases (12). Data were statistically analyzed using SPSS 21 statistical software. For reporting quantitative variables, mean and standard deviation (SD) were used and for qualitative variables, frequency and percentage were reported. To evaluate correlation and agreement rates, Pearson’s correlation test and kappa coefficient were applied. Screening performance characteristics of TQS test including sensitivity, specificity, positive and negative predictive values and positive and negative likelihood ratios were calculated with 95% confidence interval (CI) via Medical calculator. Calculation of the area under the receiver operating characteristic (ROC) curve was performed for assessing the diagnostic accuracy of TQS test. In this study, P-value less than 0.05 was considered as level of significance and ELISA was used as the reference test. 

## Results

148 patients with the mean age of 34.58 ± 15.86 years (4-86) were studied (87.8% male). Table 1 shows the baseline characteristics of the participants. Based on the findings of the TQS test, 10 (6.8%) patients had a negative serum anti-tetabulin antibody, while the number was 15 (10.1%) according to ELISA. Tetabulin was prescribed for all but 30 (20.27%) patients. 

**Figure 1:**
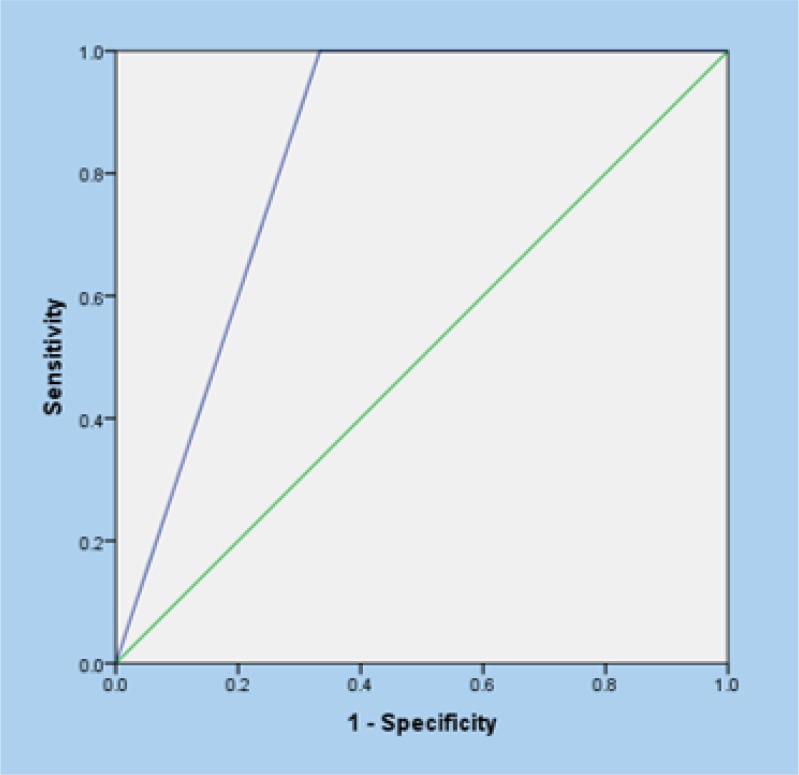
Area under the receiver operative characteristic (ROC) curve of tetanus quick stick in detection of patients with negative serum anti-tetanus IgG

The rate of overlap between results of TQS and ELISA with the patients’ history regarding complete vaccination were (r =30, p < 0.001) and (r = 0.32, p < 0.001), respectively. In addition, agreement rate between the results of TQS and ELISA was 0.78 (p < 0.001) based on calculation of kappa coefficient. Table 2 depicts the screening performance characteristics of TQS test compared to ELISA as the reference test. Area under the ROC curve of TQS for determining the serum level of anti-tetanus immunoglobulin was 0.83 (95% CI: 0.68 – 0.98) compared to ELISA (figure 1).

***Treatment cost***

In this study, the cost of injecting immunoglobulin for 118 patients was 1355.41 dollars since the cost of each tetabulin ampoule is about 11.49 dollars. Considering the true positive test results of TQS (91.7% of the cases), this cost could be reduced to 112.50 dollars, which would save 1242.91 dollars of the treatment expenses.

## Discussion

Based on the findings of the present study, TQS has good (83%) diagnostic accuracy in comparison with ELISA and considering its 100% sensitivity and negative predictive value in cases with dirty wound, it can be considered as a reliable tool for screening patients that do not need to receive anti-tetanus prophylaxis. However, considering the 66.6% specificity, it cannot be used as a confirming tool for rule in purposes.

Since being affected with the severe form of tetanus is accompanied by a high mortality rate, prophylaxis prescription for stopping the patients from being affected is very important and using tools that help identify patients in need of receiving prophylaxis can be of great help (3, 20).

**Table 1:** Baseline characteristics of the studied patients

**Variable **	**Frequency (%)**
**Age (year)**	
< 20	16 (10.8)
20 – 40	87 (58.8)
40 – 60	30 (20.3)
≥ 60	15 (10.1)
Sex	
Male	130 (87.8)
Female	18 (12.2)
**Vaccination history**	
Complete	82 (55.4)
Incomplete	66 (44.6)
**TQS** * ** result**	
Positive	138 (93.2)
Negative	10 (6.8)
**ELISA result**	
Positive	133 (89.9)
Negative	15 (10.1)
**Received immunoglobulin**	
Yes	118 (79.73)
No	30 (20.27)

* TQS: tetanus quick stick.

**Table 2:** Screening performance characteristics of tetanus quick stick (TQS) versus ELISA test in detection of patients with negative serum anti-tetanus IgG

**Characteristics**	**TQS (95 % CI)**
True positive*	133
True negative	10
False positive	0
False negative	5
Sensitivity	100 (96.50 – 100.00)
Specificity	66.66 (38.68 – 87.01)
Positive predictive value	96.37 (91.31 – 98.65)
Negative predictive value	100 (65.54 – 100.00)
Positive likelihood ratio	26.60 (11.24 – 62.93)
Negative likelihood ratio	0 (0 – NaN)

* regarding existence of anti-tetanus immunoglobulin; NaN: the calculation cannot be performed.

Results of a study on hospitalized children 1 – 9 years of age in Nigeria showed that the protective serologic level of anti-tetanus immunoglobulin using TQS and ELISA was estimated to be 45.4 and 44.7, respectively. In the study, it was shown that lack of a recent history of receiving a tetanus vaccine shot was associated with a high chance of non-protective levels of immunity. A good conformity existed between the results of ELISA and TQS and TQS test had 95.7% sensitivity, 97.6% specificity, 98% positive predictive value and 96% negative predictive value (17).

In a study aiming to determine the sensitivity and specificity of TQS as a rapid test that can be used for evaluation of the immunity condition against tetanus, it was shown that this test had a sensitivity of 88.1% and specificity of 97.6%. In addition, using this test significantly reduced the treatment costs (18).

Another study on 988 patients to compare the screening performance characteristics of TQS and ELISA revealed 76.7% sensitivity and 98% sensitivity for TQS. Overall, this study concluded that using this test in emergency settings would lead to more accurate assessments in tetanus prevention (6). In another study it was shown that the positive predictive value and specificity of TQS are 100% when compared with ELISA (19).

In the study by Stubbe et al. in Belgium to improve tetanus prophylaxis in ED it was depicted that TQS is a practical tool in ED, which significantly reduces the costs. In fact using TQS led to improved management in 56.9% patients by avoiding unnecessary treatments (14). 

Comparing TQS and clinical decisions based on vaccination history and wound type in 1658000 adult patients in 2014 indicated that using TQS is an effective and low-cost method compared to medical interview, especially in patients over the age of 65 years with wounds prone to tetanus; however, this method is considered a costly method in patients with clean wounds (3).

Results of the present study concerning the screening performance characteristics of TQS for anti-tetanus immunoglobulin are in agreement with some of the above-mentioned studies and contradict with some. The cause of these controversies in the results obtained regarding the screening performance characteristics of this test could be summarized in a few categories. First, the manufacturing company of the TQS has not been the same in all the studies and therefore, the quality of the tool could have affected its screening performance characteristics. The second point is about the studied patients. It seems that screening performance characteristics of the test vary based on wound type (tetanus prone or not). This has been clearly confirmed in the study by N'Diaye et al. in 2014 (3). In the present study, all the patients had a dirty wound and this might have caused the lower specificity estimated in this study compared to previously mentioned ones. The third point is about the reference tests in the mentioned studies, which is not the same in all of them. In some studies comparisons have been done with clinical decisions, while in others TQS has been compared with ELISA, which can be another cause for differences in their findings.

Yet overall, what all the studies agree on unanimously is reduction in costs and better management of the patients in need of receiving tetanus prophylaxis in case of using TQS. Availability, low cost and ability to do the test at the patient’s bedside are among its undeniable advantages. It seems that doing a review study and if possible, a meta-analysis for making the final decision regarding the screening performance characteristics of TQS is helpful.

***Limitations***

Including patients with dirty wounds and exclusion of patients with clean wounds might have somehow caused a selection bias in the present study. Intrinsic limitations of cross sectional studies are also another item worth mentioning in this regard.

## Conclusion:

Based on the findings of the present study, TQS has good diagnostic accuracy in comparison with ELISA and considering its 100% sensitivity and negative predictive value in cases with dirty wound, it can be considered as a reliable tool for screening patients that do not need to receive anti-tetanus prophylaxis.
